# Extending the Applicability of the Semi-experimental Approach by Means of “Template Molecule” and “Linear Regression” Models on Top of DFT Computations

**DOI:** 10.1021/acs.jpca.1c07828

**Published:** 2021-11-09

**Authors:** Alessio Melli, Francesca Tonolo, Vincenzo Barone, Cristina Puzzarini

**Affiliations:** † Scuola Normale Superiore, Piazza dei Cavalieri 7, 56126 Pisa, Italy; ‡ Dipartimento di Chimica “Giacomo Ciamician”, Università di Bologna, Via Selmi 2, 40126 Bologna, Italy

## Abstract

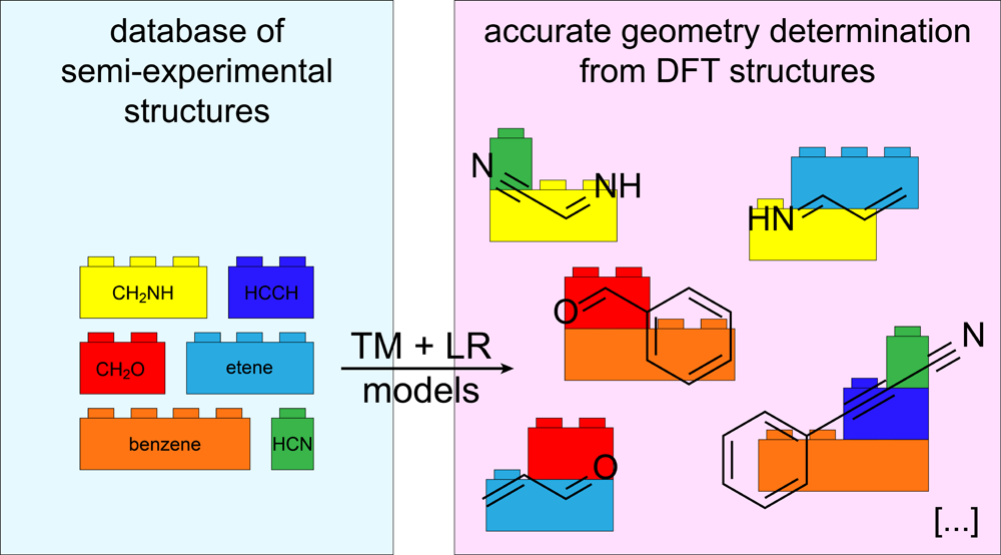

The accurate determination of equilibrium structures for isolated molecules plays a central role in the evaluation and interpretation of stereoelectronic, thermodynamic, and spectroscopic properties. For small semi-rigid systems, state-of-the-art quantum-chemical computations can rival the most sophisticated experimental results. For larger molecules, cheaper yet accurate approaches need to be defined. The double-hybrid rev-DSD-PBEP86 functional already delivers remarkable results that can be further improved by means of a “Lego brick” model. This is based on the idea that a molecular system can be seen as formed by different fragments (the “Lego bricks”), whose accurate semi-experimental (SE) equilibrium geometries are available. The template molecule (TM) approach can be used to account for the modifications occurring when going from the isolated fragment to the molecular system under investigation, with the linear regression (LR) model employed to correct the linkage between the different fragments. The resulting TM-SE_LR approach has been tested with respect to available SE equilibrium structures and rotational constants. Indeed, the latter parameters straightforwardly depend on the equilibrium geometry of the system under consideration. The main outcome of our study is the reliability, robustness, and accuracy of this novel approach. The molecular systems considered for benchmarking the TM-SE_LR scheme are those formally issued from addition/elimination reactions of nucleophilic unsaturated radicals (e.g., CN, C_2_H, and phenyl) to alkenes, imines, and aldehydes, whose rotational spectra have been investigated, but accurate structural determinations are not yet available.

## Introduction

The accurate determination of molecular structures is one of the main aims in many areas of chemistry. In physical chemistry, the prediction and interpretation of structural properties and dynamic behavior of molecules are a prerequisite for a deeper understanding of their stability and chemical reactivity.^1−10^ In the field of molecular spectroscopy, there is a strong relationship between the experimental outcomes and the structure of the molecular system.^11−14^ Therefore, computational spectroscopy investigations often start from structural determinations. In particular, in rotational spectroscopy, the equilibrium geometry provides the major contribution to the leading terms of this technique, the rotational constants.^2,6,11^ While it is seldom straightforward to derive the molecular structure from experimental information, with quantum chemistry being often required to either support or complement such determinations, state-of-the-art computational methodologies are able to provide very accurate equilibrium structures.^6,7,15,16^ Indeed, in the absence of strong non-dynamical correlation effects, composite schemes rooted in the coupled-cluster model are currently able to evaluate structural parameters with an accuracy of 0.001 Å and 0.1° for bond lengths and angles, respectively.^6,17−23^ However, despite the improvement in hardware and software technologies^24,25^ and the increasing availability of composite schemes at reduced computational costs (e.g., the so-called “cheap” scheme and its variants),^22,26^ accurate geometry optimizations are still out of range for large molecular systems due to the extremely unfavorable scaling of accurate quantum-chemical methods with the number of degrees of freedom.

A viable route is offered by the interplay of the experiment and theory in the field of rotational spectroscopy (see, e.g., refs (6),^27^–^32^). Within the so-called semi-experimental (SE) approach,^6,33^ equilibrium structural parameters are derived from a least-squares fit of the SE equilibrium rotational constants (*B*_e_^SE^) for different isotopologues. The *B*_e_^SE^ values are obtained by correcting the experimental vibrational ground-state rotational constants^a^ (*B*_0_^exp^) for computed vibrational contributions (Δ*B*_vib_^calc^)^33^
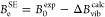
1

The accuracy of this approach is well established,^27^ and its application led to the definition of a database of SE equilibrium structures of small- to medium-sized semi-rigid molecules (hereafter denoted as the SE database).^29,30^

The main drawback of the SE approach is the number of experimental data required for a complete structural characterization. The greater the molecule is, the larger the number of isotopologues to be investigated becomes. Furthermore, even if the number of available *B*_0_^exp^ constants exceeds that of the geometrical parameters to be determined, a balanced fit requires data for the isotopic substitutions involving all nuclei.^14^ This becomes exceedingly difficult when the molecular size and topological complexity increase. If the number of available isotopically substituted species is not sufficient to allow a robust evaluation of all structural parameters, the lack of information can affect both the quality of the fit and their accuracy and reliability. In these cases, to avoid biased results, some geometrical parameters (i.e., those whose “experimental information” is missing) are kept fixed in the fitting procedure, usually relying on computational determinations. However, if the level of theory employed is not sufficiently high, the results of the fit and their accuracy might be unsatisfactory.^6^ In this respect, powerful way-outs are offered by the template molecule (TM)^30^ and/or linear regression (LR) approaches.^29^

The TM approach has been introduced to extend the size of molecular systems amenable to accurate molecular structure determinations. It is based on the idea that, if an accurate equilibrium geometry is available (either experimentally or computationally) for a smaller molecule identical to one of the system’s moieties, it can be used to provide the corresponding geometrical parameters of the system under study, corrected by the differences between predictions calculated for the moieties and the system. Alternatively, the LR approach can be employed to improve the computed geometries. Indeed, within this approach, a given structural parameter is corrected by a term previously obtained from the linear regression of the fit of semi-experimental values versus the computed counterparts (at the level of theory considered) for a large set of molecules. The LR corrections are available for the most common bond distances and angles and have been collected in a database (hereafter denoted as the LR database) for several combinations of density functionals and basis sets.^29,30,34^

The idea presented in this work is to combine the TM and SE approaches, also relying on the contribution of LR corrections, with the aim of obtaining highly accurate equilibrium structures at a very reduced computational cost. The so-called TM-SE approach is based on identifying, in the molecule under study, fragments whose structures are available in the SE database mentioned above and using them to accurately determine its equilibrium geometry, possibly employing the LR approach for inter-fragment parameters (thus leading to the TM-SE_LR approach).

Among the different classes of molecular systems whose accurate structures are not yet available, one cannot overlook those issued from addition/elimination reactions of nucleophilic unsaturated radicals (e.g., CN, C_2_H, and phenyl) to alkenes, imines, and aldehydes. As a matter of fact, addition of free radicals to double bonds has been studied at length because of its interest in several fields ranging from organic synthesis to atmospheric chemistry and astrochemistry (see refs (35−38) and references therein). In particular, the products of addition/elimination of free radicals to alkenes and aldehydes are well known, but accurate structures of some of them are not yet available. The situation is even more involved for imines, which—despite their low-stability under normal terrestrial conditions—play a central role in several processes, leading to the formation of the so-called complex organic molecules in the inter- and circumstellar medium (see refs (38−41) and references therein). Overall, all products of the reactions mentioned above can be seen as formed by two well-defined moieties formally linked by a single bond between two sp^2^ and/or sp carbon atoms. Among the possible products, we have chosen the systems forming a suitable playground for testing the proposed TM-SE and TM-SE_LR approaches.

In the next section, the methodology is described with all details. Then, once the set of molecular systems has been introduced, the results are reported, and the validation of our approach is discussed. Examples of applications demonstrating the approach’s possible extension to larger systems are also provided. Finally, concluding remarks summarize the main outcomes of this work.

## Methodology

The TM-SE(_LR) approach where, for a given molecule, suitable molecular fragments are seen as “Lego bricks” is set up as follows:

- For the “Lego bricks”, we resort to the TM approach, which—as mentioned in the Introduction—is based on identifying, within the molecule, “known” fragments that belong to a smaller system for which a highly accurate equilibrium geometry is available. These fragments are the TMs and are used to determine accurately the corresponding structural parameters of the larger molecular systems (*r*_e_(lms))
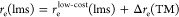
2where Δ*r*_e_(TM) is defined as
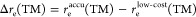
3*r*_e_^low-cost^ is the geometrical parameter of interest calculated at the same level of theory for both the molecule under consideration (lms) and the TM, the level depending on the size of the molecule to be characterized (usually a method rooted in density functional theory, DFT). A recent benchmark study^42^ showed that the double-hybrid rev-DSD-PBEP86 functional^43^ in conjunction with the jun-cc-pVTZ basis set^44,45^ already provides rather accurate structures. Hereafter, this level of theory, shortly denoted as revDSD, will be employed for the *r*_e_^low-cost^(lms) and *r*_e_^low-cost^(TM) of eqs 2 and 3, respectively.

- Once we have the “Lego bricks”, we need to put them together, the arising question being how to connect them. For this task, whenever possible, we resort to the LR approach for improving the accuracy of DFT determinations of the inter-fragment parameters. For them, the LR approach replaces the Δ*r*_e_(TM) correction in eq 2 with an estimate (Δ*r*_e_(LR)) based on a linear regression model
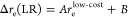
4Therefore, the corrected parameter is given by
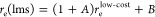
5

The *A* and *B* coefficients only depend on the atomic numbers of the involved atoms and were obtained by a statistical analysis of a large number of molecules. In the present context, we employ the corrective factor for the C–C (*A* = −0.0067 and *B* = 0.0069) bond length evaluated using the revDSD model and obtained from a study employing nearly 100 semi-experimental values.

If only step 1 is retained, with the linkage parameters thus kept fixed at the revDSD level, the TM-SE model is defined. The TM-SE approach is so denoted because the TMs are taken from the SE database mentioned in the Introduction. The combination of the steps 1 and 2 leads instead to the so-called TM-SE_LR approach. While in the following, we demonstrate the accuracy of the equilibrium structures “built” using the TM-SE and TM-SE_LR models, here, we note that if this approach is used to support a rotational spectroscopy study, once the latter is completed, the SE approach can be employed to refine the revDSD or LR linkage parameters, which are usually the most sensitive structural parameters.

**Figure 1:**
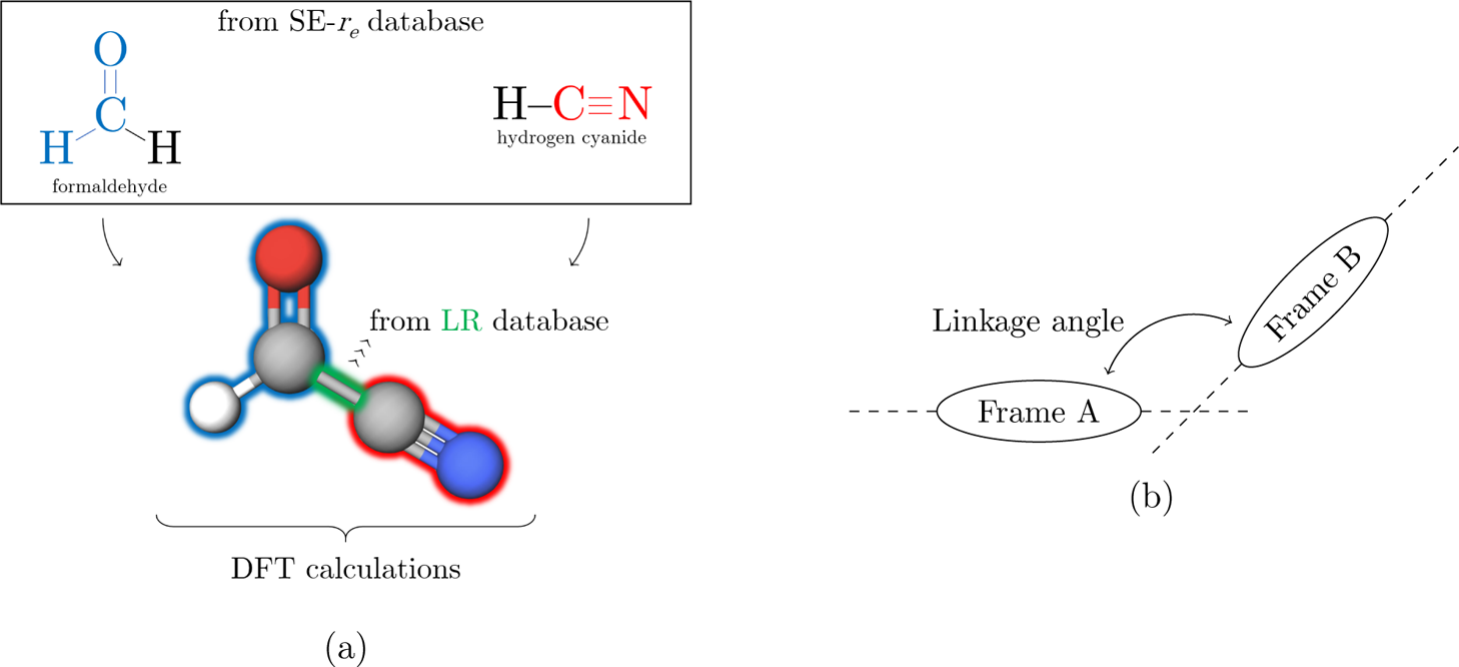
(a) Graphical description of the TM-SE_LR approach. (b) Definition of the linkage angle.

A graphical representation of the TM-SE_LR approach is provided in Figure 1a. The example considered in this figure is formyl cyanide. In this molecule, two molecular fragments can be envisaged: the CN and HCO moieties. DFT geometry optimizations are carried out for formyl cyanide and for the TMs, namely, HCN and formaldehyde. The data available in the SE database are then used to correct the structural parameters of the CN and HCO moieties of formyl cyanide (see eqs 2 and 3). Finally, the DFT C–C bond distance connecting the two “Lego bricks” is corrected using the scaling factors available in the LR database.

To test the accuracy of the equilibrium structures derived using the TM-SE(_LR) approach, one can compare them with SE equilibrium geometries already available in the literature. However, this is not the best option, the TM-SE approach being designed for those cases in which the standard SE approach cannot be applied. Since rotational constants are strongly connected to molecular structures and they are experimentally determined with great accuracy,^2,6,11,27^ they are the perfect means for our test. According to eq 1, to move from the ground-state to the equilibrium rotational constants and vice versa, we need the vibrational corrections, Δ*B*_vib_. In the framework of vibrational perturbation theory to the second order (VPT2),^46^ these are expressed as
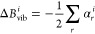
6where the α_*r*_^*i*^ constants are the vibration–rotation interaction constants, *i* denotes the inertial axis, and the sum runs over all *r* vibrational modes. The evaluation of the α_*r*_^*i*^ values requires anharmonic force field calculations,^14,23,27,28,32^ which—in the present work—have been carried out using the global-hybrid B3LYP functional^47,48^ in conjunction with the partially augmented double-ζ jun-cc-pVDZ basis set. Hereafter, this level of theory is shortly referred to as B3. Although revDSD spectroscopic parameters are usually more accurate than the B3 counterparts,^42^ the Δ*B*_vib_ contributions benefit from a fortuitous but quite general error compensation, which allows the use of cheaper B3 computations.

All DFT calculations incorporate the D3 scheme^49^ for the treatment of dispersion effects combined with the Becke-Johnson (BJ) damping function.^50^ Throughout this work, all computations have been performed using the Gaussian16 suite of programs.^51^

### Dataset

**Figure 2:**
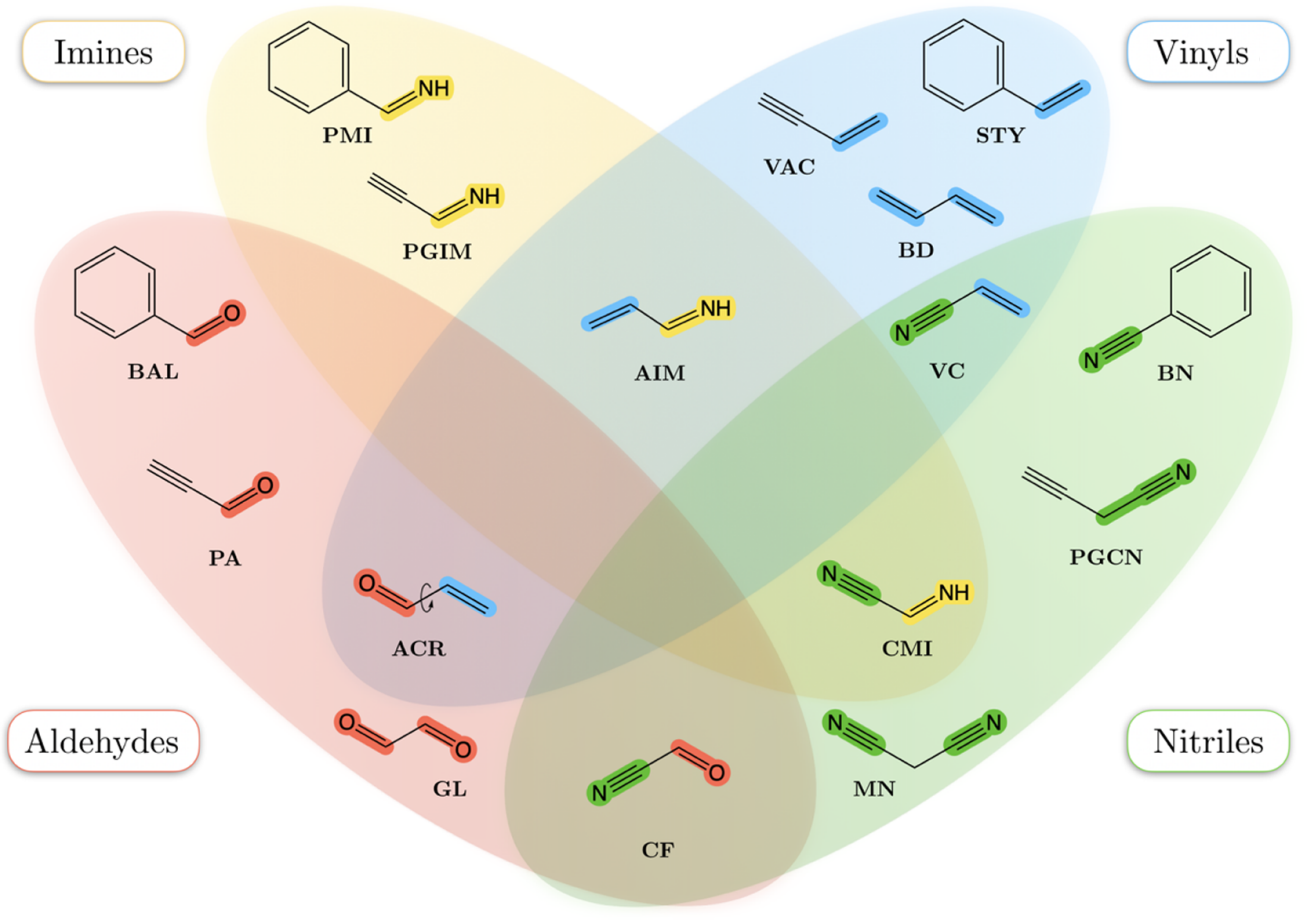
Dataset: molecules grouped according to their moieties, “Lego” bricks.

Before reporting and discussing the results, we need to introduce and describe the dataset employed in our study. The targeted molecules are listed in Figure 2 and have been selected following two main criteria: (1) the rotational constants of the main isotopic species are available and (2) each molecule can be envisaged as formed by two fragments, whose SE equilibrium structures are available. Going into detail, the dataset is composed of 16 species (21 molecules if isomers are considered) that result from the combination of seven fragments, namely, methanimine (CH_2_NH), formaldehyde (H_2_CO), hydrogen cyanide (HCN), acetylene (C_2_H_2_), ethene (C_2_H_4_), acetonitrile (CH_3_CN), and benzene (C_6_H_6_). The SE equilibrium structures of all these fragments are available in the SE database,^29,30^ the only exception being methanimine and acetonitrile, whose SE equilibrium geometries are taken from refs (52) and (53), respectively. The SE equilibrium geometries employed in this work are collected in Table S1.

The structural patterns of the chosen molecules allow their classification into four distinct and partially overlapping ensembles, depicted in Figure 2, which shows (also highlighted by different colors) the “Lego bricks” within the selected molecules. The ensemble of imines, that is, species containing the −C=NH terminal group (in yellow in Figure 2), consists of four molecules: propargylimine (PGIM), phenylmethanimine (PMI), cyanomethanimine (CMI), and allylimine (AIM). The orientation of the H atom of the −CNH moiety with respect to the other fragments of the molecule leads to the *Z* (*cis*) and *E* (*trans*) isomers. In this study, all the eight structures have been considered, the experimental rotational constants being retrieved from the reference list provided in Table 1. By replacing the imine moiety with the aldehydic −HC=O group, we obtain four molecules characterized well from an experimental point of view (see Table 1), namely, benzaldehyde (BAL), propriolaldehyde (PA), acrolein (ACR; both the *cis* and *trans* isomers), and cyanoformaldehyde (CF). The connection of two aldehydic fragments also leads to the formation of glyoxal (GL; only *trans*). They all have been collected in the red ensemble in Figure 2. Four molecules, namely, styrene (STY), vinylacetylene (VAC), 1,3-butadiene (BD; only the *trans* form), and vinyl cyanide (VC), have been selected to incorporate the vinyl −CHCH_2_ group in the studied set of molecules, this being the light-blue set. Finally, three molecules containing the cyano −CN group, namely, benzonitrile (BN), malononitrile (MN), and propalgylcyanide (PGCN), have also been considered. The nitrile color code is green.

**Table 1:**
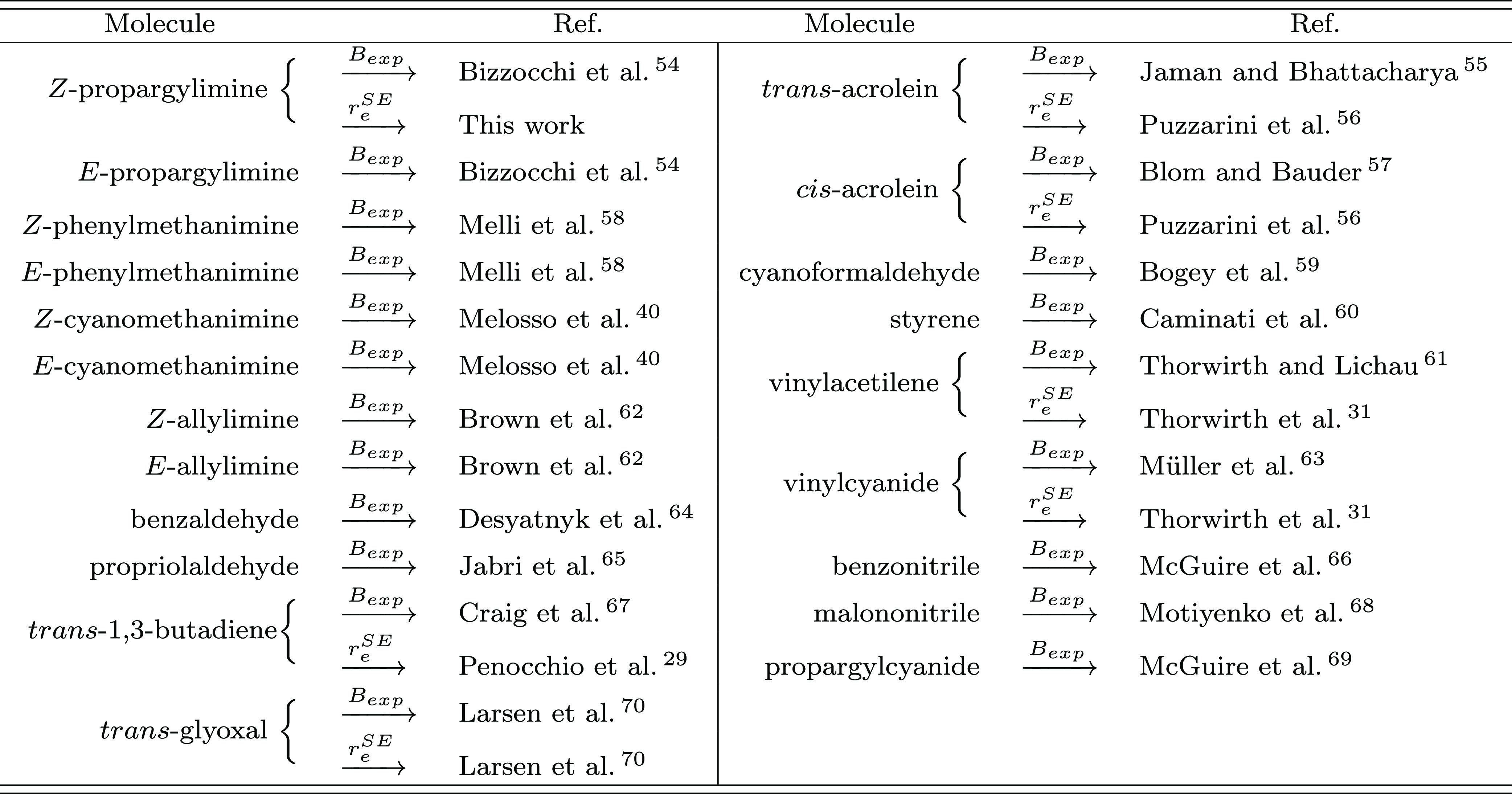
Reference Studies Reporting the Experimental Ground-State Rotational Constants (*B*_0_^exp^) and Available SE Equilibrium Structures (*r*_e_^SE^) for the Dataset Molecules

With the exception of MN and PGCN, all molecules present, as a linkage bond, a single CC bond connecting two unsaturated systems, and thus, non-negligible conjugation effects are expected. Therefore, the selected molecules provide a challenging test suite to validate the TM-SE model and, especially, the TM-SE_LR approach. Some molecular species present a certain degree of flexibility, which is another challenging aspect for our strategy. In particular, the two non-conjugated molecules, that is, MN and PGCN, should be good test cases with their C−C−C frame.

In Table 1, together with the list of the reference studies for the selected molecules, the references for the available SE equilibrium structures are also reported.

## Results and Discussion

Let us start our discussion by comparing the molecular structures issued from the TM-SE and TM-SE_LR approaches with the SE equilibrium geometries (*r*_e_^SE^) available in the literature for VAC, VC, *trans*-acrolein (*t*-ACR), *cis*-acrolein (*c*-ACR), *trans*-1,3-butadiene (*t*-BD), *trans*-glyoxal (*t*-GL), and *Z*-propargylimine (*Z*-PGIM). The *r*_e_^SE^ structures of *t*-ACR and *c*-ACR have been taken from ref (56) and those of *t*-GL have been taken from ref (70); they are also available in the SE database^29^ together with the *r*_e_^SE^ of *t*-BD. Those of VC and VAC were evaluated in ref (31) and that of *Z*-PGIM has been purposely determined in this work using the data from refs (54) and (71). For *Z*-PGIM, the availability, in addition to the rotational constants of the parent isotopologues, of the data for the three ^13^C isotopic species and two deuterated variants (at the −NH and −CCH sites) has enabled a reliable, even if partial, determination of the SE equilibrium structure.

**Figure 3:**
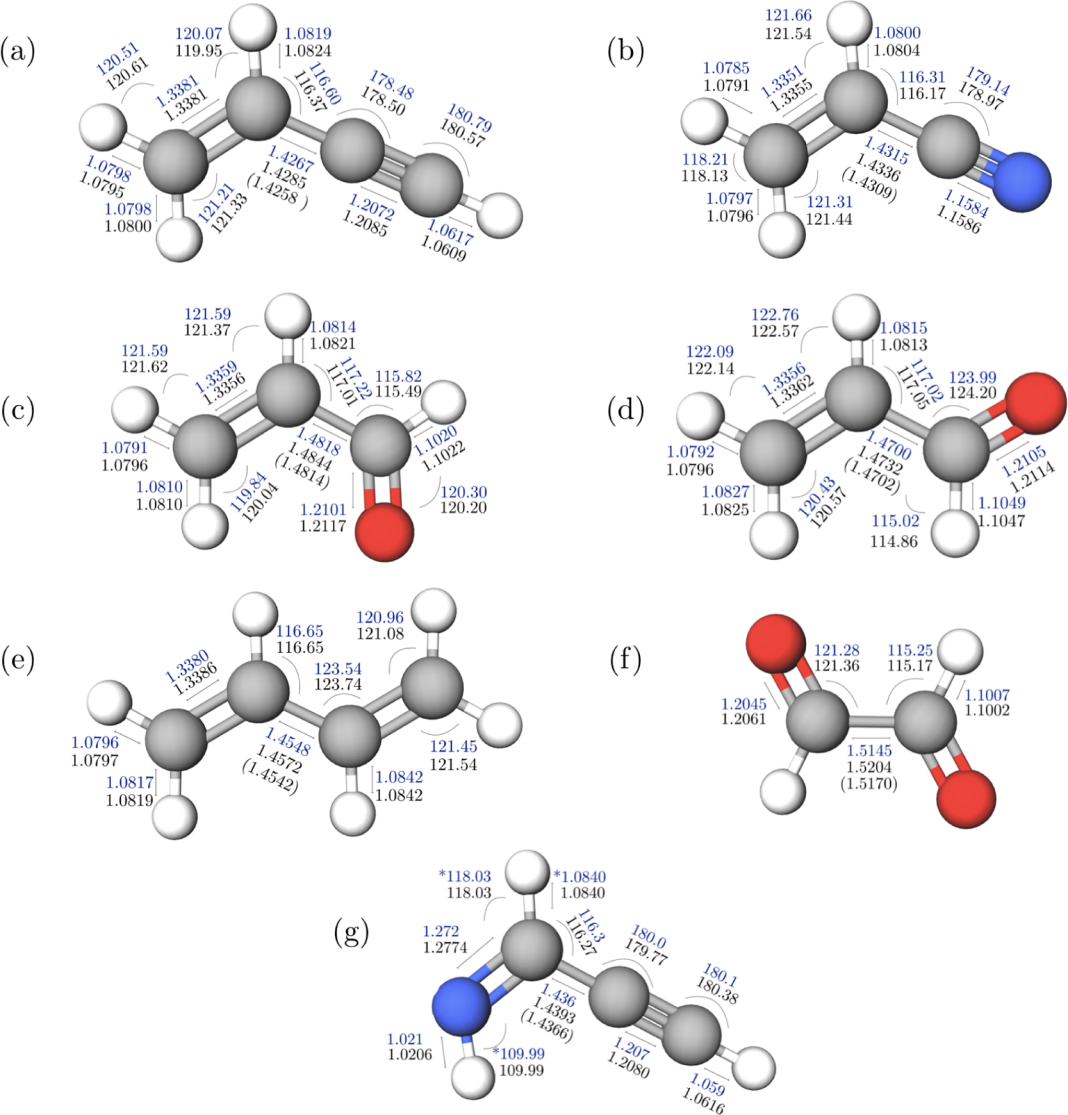
Molecular structures (bond lengths in angstrom and angles in degrees) of vinylacetylene (a), vinyl cyanide (b), *trans*-acrolein (c), *cis*-acrolein (d), *trans*-1,3-butadiene (e), *trans*-glyoxal (f), and *Z*-propargylimine (g). The TM-SE values are in black (for the C–C linkage bond, the TM-SE_LR value is also reported); SE parameters (errors are available in the Supporting Information, Table S2) are in blue (the asterisk denotes parameters kept fixed at the TM-SE value; the number of decimal places depends on the accuracy; see Table S2).

Figure 3 shows the molecular structures of the seven test cases, also providing the comparison between the TM-SE_LR and SE equilibrium geometries. These are also collected in Table S2 of the Supporting Information together with the uncertainties affecting the *r*_e_^SE^ structures and the revDSD structural parameters. The fragments employed in the TM-SE are evident: ethylene and acetylene for VAC, ethylene and hydrogen cyanide for VC, ethylene and formaldehyde for *t*-ACR and *c*-ACR, ethylene for *t*-BD, formaldehyde for *t*-GL, and methanimine and acetylene for *Z*-PGIM. Their revDSD and SE structural parameters are collected in Table S1 of the Supporting Information. From Figure 3, a very good agreement between the TM-SE(_LR) and *r*_e_^SE^ geometries is apparent. It is noted that the deviations are in the order of 0.001 Å for bond lengths and 0.1° for angles. There are only a very few exceptions showing discrepancies larger than 0.001 Å (but always within 2 mÅ), and these can be ascribed to conjugative effects. While the revDSD level already provides good results (deviations in the order of +0.003 Å for bond lengths and |0.2|° for angles; see Table S2 of the Supporting Information), the improvement provided by the TM-SE approach is apparent. The only somewhat uncertain comparison concerns *Z*-PGIM, for which the experimental data are not sufficient for a complete structural determination. At this point, it is interesting to check how the small discrepancies observed in the structural parameters reflect on the rotational constants, the results being reported in Table 2. Indeed, rotational constants are extremely sensitive to the molecular structure. In fact, variations of 0.001 Å in the bond distances and 0.1° in valence angles can lead to changes up to ±50 MHz for the *A* constant and ±15 MHz for *B* and *C*. This issue is very important because the first application we have in mind is the prediction of the rotational spectra of “unknown” molecules with a very good accuracy at a limited computational cost.

**Table 2:** Comparison of Computed Equilibrium Rotational Constants (in MHz) with the SE Counterparts for Vinylacetylene, Vinyl Cyanide, *trans*-Acrolein, *cis*-Acrolein, *trans*-1,3-Butadiene, *trans*-Glyoxal, and *Z*-Propargylimine^a^

	revDSD	TM-SE	TM-SE_LR	TM-SE_LR + corr^b^	SE(*r*_e_^SE^)	SE(*B*_e_^SE^)-B3^c^	SE(*B*_e_^SE^)-CC^d^
vinylacetylene							
*A*_e_	50611.7 (+0.39%)	50741.1 (+0.65%)	50751.6 (+0.67%)	50552.9 (+0.28%)	50413.9	50602.3 (+188.4)	50425.4 (+11.5)
*B*_e_	4730.2 (−0.59%)	4738.2 (−0.42%)	4745.9 (−0.26%)	4752.6 (−0.12%)	4758.2	4759.4 (+1.2)	4758.0 (−0.2)
*C*_e_	4325.9 (−0.50%)	4333.5 (−0.33%)	4340.1 (−0.18%)	4344.2 (−0.08%)	4347.8	4350.1 (+2.3)	4347.6 (−0.2)
vinyl cyanide							
*A*_e_	50115.1 (+0.35%)	50250.1 (+0.62%)	50262.7 (+0.64%)	50071.7 (+0.27%)	49939.0	49893.3 (−45.6)	49943.9 (+4.9)
*B*_e_	4955.5 (−0.64%)	4967.0 (−0.41%)	4975.5 (−0.24%)	4982.8 (−0.09%)	4987.3	4986.5 (−0.8)	4987.3 (+0.0)
*C*_e_	4509.6 (−0.55%)	4520.2 (−0.31%)	4527.4 (−0.15%)	4531.9 (−0.06%)	4534.4	4533.3 (−1.1)	4534.5 (+0.1)
*trans*-acrolein							
*A*_e_	47786.8 (−0.15%)	47934.4 (+0.15%)	47996.9 (+0.28%)	47899.1 (+0.08%)	47860.9	47857.9 (−2.1)	47836.7 (−23.3)
*B*_e_	4660.4 (−0.58%)	4667.9 (−0.42%)	4675.5 (−0.26%)	4680.3 (−0.16%)	4687.8	4685.0 (−2.8)	4688.2 (+0.4)
*C*_e_	4246.3 (−0.55%)	4253.7 (−0.37%)	4260.5 (−0.21%)	4263.7 (−0.14%)	4269.6	4267.9 (−1.7)	4270.2 (+0.6)
*cis*-acrolein							
*A*_e_	22938.3 (+0.19%)	23009.8 (+0.50%)	23010.0 (+0.50%)	22971.9 (−0.10%)	22895.7	22896.4 (+0.7)	22892.8 (−2.9)
*B*_e_	6228.5 (−1.12%)	6235.5 (−1.01%)	6250.2 (−0.78%)	6263.0 (−0.57%)	6299.1	6295.8 (−3.2)	6303.3 (+4.2)
*C*_e_	4898.4 (−0.83%)	4906.0 (−0.69%)	4915.1 (−0.50%)	4921.3 (−0.38%)	4940.0	4939.4 (−0.6)	4943.4 (+3.4)
*trans*-1,3-butadiene							
*A*_e_	42069.0 (−0.10%)	42190.6 (+0.19%)	42239.2 (+0.30%)	42123.9 (+0.03%)	42111.2	42127.1 (+16.0)	
*B*_e_	4441.7 (−0.46%)	4445.0 (−0.38%)	4451.8 (−0.23%)	4459.7 (−0.05%)	4462.1	4460.6 (−1.4)	
*C*_e_	4017.5 (−0.42%)	4021.3 (−0.33%)	4027.3 (−0.18%)	4032.7 (−0.05%)	4034.6	4034.3 (−0.3)	
*trans*-glyoxal							
*A*_e_	55888.5 (−0.51%)	56092.7 (−0.15%)	56183.9 (+0.01%)	55973.2 (−0.36%)	56177.3	55955.7 (−221.6)	56007.1 (−170.2)
*B*_e_	4781.5 (−0.80%)	4792.6 (−0.57%)	4802.1 (−0.37%)	4811.6 (−0.17%)	4819.9	4821.0 (+1.1)	4819.9 (0.0)
*C*_e_	4404.7 (−0.68%)	4415.4 (−0.53%)	4423.9 (−0.34%)	4430.7 (−0.19%)	4439.1	4439.0 (−0.1)	4439.0 (−0.1)
*Z*-propargylimine							
*A*_e_	54707.3 (−0.57%)	54894.9 (−0.23%)	54904.5 (−0.21%)	54690.0 (−0.61%)	55022.9	55040.7 (+17.8)	
*B*_e_	4850.0 (−0.68%)	4858.9 (−0.50%)	4867.2 (−0.33%)	4873.9 (−0.19%)	4883.4	4883.2 (−0.2)	
*C*_e_	4455.1 (−0.68%)	4463.8 (−0.48%)	4470.8 (−0.33%)	4475.1 (−0.23%)	4485.5	4484.2 (−1.3)	

a The relative differences with respect to SE(*r*_e_^SE^) are reported within parentheses.

b Correction of −0.2° on the linkage angle. Referring to Figure 1b, the minus sign denotes a lowering of the linkage angle.

c SE(*B*_e_^SE^) using vibrational corrections at the B3 level. The difference SE(*B*_e_^SE^) – SE(*r*_e_^SE^) is reported within parentheses.

d SE(*B*_e_^SE^) using vibrational corrections at the fc-CCSD(T)/cc-pVTZ level [from ref (31) for VAC and VC, from ref (56) for ACR (*trans* and *cis*), and from ref (70) for *t*-GL]. The difference SE(*B*_e_^SE^) – SE(*r*_e_^SE^) is reported within parentheses.

In Table 2, the equilibrium rotational constants straightforwardly derived from the *r*_e_^SE^ structures are denoted as SE(*r*_e_^SE^). For comparison purposes, those derived from the experimental ground-state rotational constants corrected for the vibrational contributions at the B3 level are also reported, these being denoted as SE(*B*_e_^SE^)-B3. For VAC, VC, *t*-ACR, *c*-ACR, and *t*-GL, the SE(*B*_e_^SE^) values obtained using vibrational corrections at the fc-CCSD(T)/cc-pVTZ^44^ level available in the literature^30,31^ are also given (SE(*B*_e_^SE^)-CC). CCSD(T)^72^ denotes the coupled-cluster single and double approximation augmented by a perturbative treatment of triples, and fc stands for the frozen-core approximation. The comparison between these two sets of SE *B*_e_ constants is crucial because for the other molecules of the dataset, the performance of the TM-SE(_LR) approach will be based on the latter type of SE *B*_e_ values. In principle, the two routes should lead to equivalent results. However, we note that vibrational corrections at the CCSD(T) level tend to provide a better agreement, with the average discrepancy being, in relative terms, ∼0.04%. When B3 vibrational corrections are considered, the differences are a bit larger, but the mean deviation is still as low as 0.06%. We note a few large discrepancies from the SE(*r*_e_^SE^) values, all for the *A* constant. When considering the B3 vibrational corrections, the largest deviations are 0.37% for VAC and 0.40% for *t*-GL. In the case of CCSD(T) vibrational corrections, the only outlier is observed for *t*-GL, the deviation being 0.30%. If we exclude these data, the relative averaged discrepancies from SE(*r*_e_^SE^) reduce to 0.02% for CCSD(T) and 0.03% for B3. This comparison provides a quantification of the systematic error affecting our analysis. Conservatively, we consider that the SE equilibrium rotational constants employed in the analysis of the dataset (i.e., those obtained with B3 vibrational corrections) are affected by a systematic uncertainty well within 0.1%. At this point, the accuracy of the SE equilibrium structure deserves a note. While the limited accuracy of the B3 vibrational corrections affects, even if marginally, the derived SE equilibrium rotational constants, the effects are entirely negligible on the structural determination, as demonstrated well by the literature on this topic; for example, see refs (27) and (30).

Before discussing the results of Table 2, it should be pointed out that the accuracy reached by the TM-SE and TM-SE_LR models can only be approached by applying expensive accurate composite schemes based on coupled-cluster theory.^2,5,73,74^ An example is provided by the experimental investigation on the rotational spectrum of propargylimine, which also led to its detection in the interstellar medium.^54^ In that work, the spectroscopic study was supported by quantum-chemical calculations, with equilibrium rotational constants obtained by means of the so-called “CCSD(T)/CBS + CV” composite scheme,^19,20^ whose accuracy has been tested in refs (2) and (73). This composite scheme exploits the CCSD(T) method extrapolated to the complete basis set (CBS) limit (within the fc approximation) and incorporates the core-valence (CV) correlation correction. The CCSD(T)/CBS + CV *B*_e_ constants are 54525.7, 4876.6, and 4476.3 MHz, which deviate from the SE counterparts by 0.90, 0.14, and 0.21%, respectively, to be compared with those issuing from the TM-SE_LR model, which differ instead by 0.21, 0.33, and 0.33% (see Table 2). Another example is provided by ACR, which points out how accurate the CCSD(T)/CBS + CV composite scheme can be. In ref (56), this approach was employed in the geometry optimization of *t*- and *c*-ACR. The corresponding equilibrium rotational constants (22915.2, 6301.4, and 4942.3 MHz for *c*-ACR and 47870.0, 4689.3, and 4270.9 MHz for *t*-ACR) were found to deviate, on average, by only 0.04% from the SE(*r*_e_^SE^) values.

From the comparisons of Table 2, we note that—for all test cases—the deviations are small at all the levels considered, with an overall improvement when moving from revDSD to TM-SE. A further improvement is noted by resorting to the TM-SE_LR approach. In more detail, the absolute mean deviations from the SE(*r*_e_^SE^) values are 0.5% at the revDSD level, 0.4% for TM-SE, and 0.3% for TM-SE_LR. In several cases, as already noted in the comparison between SE(*r*_e_^SE^) and SE(*B*_e_^SE^) and well-known in the literature (see, e.g., ref (74)), the agreement for the *A* constant is the most critical among the three rotational constants. Indeed, it worsens when moving from the revDSD level to the TM-SE approach, with the LR corrections being unable to improve this discrepancy. An inspection of the revDSD, SE, and TM-SE structures revealed that this is caused by very small structural modifications, and in detail, it seems to be due to missing LR corrections for linkage angles (see Figure 1b for the definition). Actually, the LR terms are available for angles, but the inter-fragment angles tend to behave differently. They somewhat resemble the inter-molecular angles in non-covalent complexes. While this might be a sort of limitation in predicting the rotational constants and the corresponding spectrum of an “unknown” molecule with high accuracy, once the latter is assigned and analyzed, the experimental rotational constants of just one isotopic species are sufficient to effectively correct the linkage parameters and lead to an equilibrium structure of great accuracy. For example, if we consider *Z*-CMI and refine the revDSD linkage parameters (the C–C distance and the corresponding angle) using the experimental rotational constants of the main isotopic species (the only ones investigated so far), the discrepancies with respect to the SE *B*_e_^SE^ values reduce to −0.02, 0.001, and 0.002% for *A*, *B*, and *C*, respectively, with these being −0.21, −0.49, and −0.47%, respectively, at the TM-SE level (see Table S3). Even if we only fit the C–C linkage bond, the resulting discrepancies are extremely good: −0.15, 0.006, and 0.004%, respectively.

For the seven molecules under consideration, tests have been carried out in order to understand if a systematic corrective term can be applied to the linkage angle (see Figure 1b). The outcome of these tests (see the fourth column of Table 2) suggests that indeed, a lowering of 0.2° improves the agreement of the TM-SE_LR rotational constants with respect to the SE ones, with the only exception of the A rotational constant of *t*-GL and *Z*-PGIM. Such an improvement leads to a reduction in the absolute mean deviation to 0.2%. Interestingly, for *Z*-PGIM, an additional correction (−0.002 Å) to the C–C LR corrective term leads to an improvement for all rotational constants: 54911.6 MHz (−0.20%), 4873.3 MHz (−0.21%), and 4476.1 MHz (−0.21%). These results seem to suggest that the LR correction is not entirely able to recover conjugative effects due to double and triple bonds connected to the C–C single linkage. Such a limitation is also expected for *c*-ACR, for which the deviations on *B* and *C* are rather significant for all levels of theory, for example, from −1.12% for B at the revDSD level to −0.78% for TM-SE_LR. While the additional corrective term works well for all imines, we anticipate that the “–0.2° correction” extended to the entire dataset works fine only for the molecules containing the vinyl frame (see Table S3).

As mentioned in the Methodology section, the TM-SE and TM-SE_LR approaches have been extended to the dataset introduced above. The results are collected in Table S3 of the Supporting Information, where the revDSD, TM-SE, and TM-SE_LR equilibrium rotational constants are compared with the SE(*B*_e_^SE^) constants obtained from the experimental ground-state rotational constants (*B*_0_^exp^) and the B3 vibrational corrections. The *B*_0_^exp^ constants and the corresponding Δ*B*_vib_^B3^ corrections are reported in Table S3, which also collects the TM-SE_LR + corr results, evaluated as explained and addressed above.

From the inspection of Table S3, results in line with those of Table 2 are noted. In almost all cases, the revDSD level provides relative deviations with respect to the *B*_e_^SE^ values within 1%. The use of the TM-SE approach improves the agreement in almost all cases, with an average relative deviation of 0.3%. A further improvement is noted once the LR corrections are considered (TM-SE_LR approach), the average relative deviation reducing to 0.2%. Based on the accuracy of the SE equilibrium rotational constants discussed above, the relative error of the TM-SE_LR rotational constants can be—on average—as low as 0.1%. Such a good agreement on the SE *B*_e_ constants implies that the TM-SE_LR structures are highly accurate, which means average deviations smaller than 0.001 Å for bond lengths and 0.1° for angles. Finally, we note that the more rigid the molecule is, the better the TM-SE approach works, as demonstrated—for example—by phenylmethanimine and benzonitrile.

### Rotational Spectrum of Phenylmethanimine

As mentioned above, the TM-SE approach is particularly accurate for rigid molecular systems. To demonstrate this outcome, we applied it to the simulation of the rotational spectrum of *E*- and *Z*-PMI. For this purpose, the ground-state rotational constants have been predicted by adding the B3 vibrational corrections to the revDSD and TM-SE_LR equilibrium values (according to eq 1). To complete the set of the required spectroscopic parameters, namely, the quartic centrifugal-distortion and nitrogen quadrupole-coupling constants, we refer to the computational results reported in ref (58).

**Figure 4:**
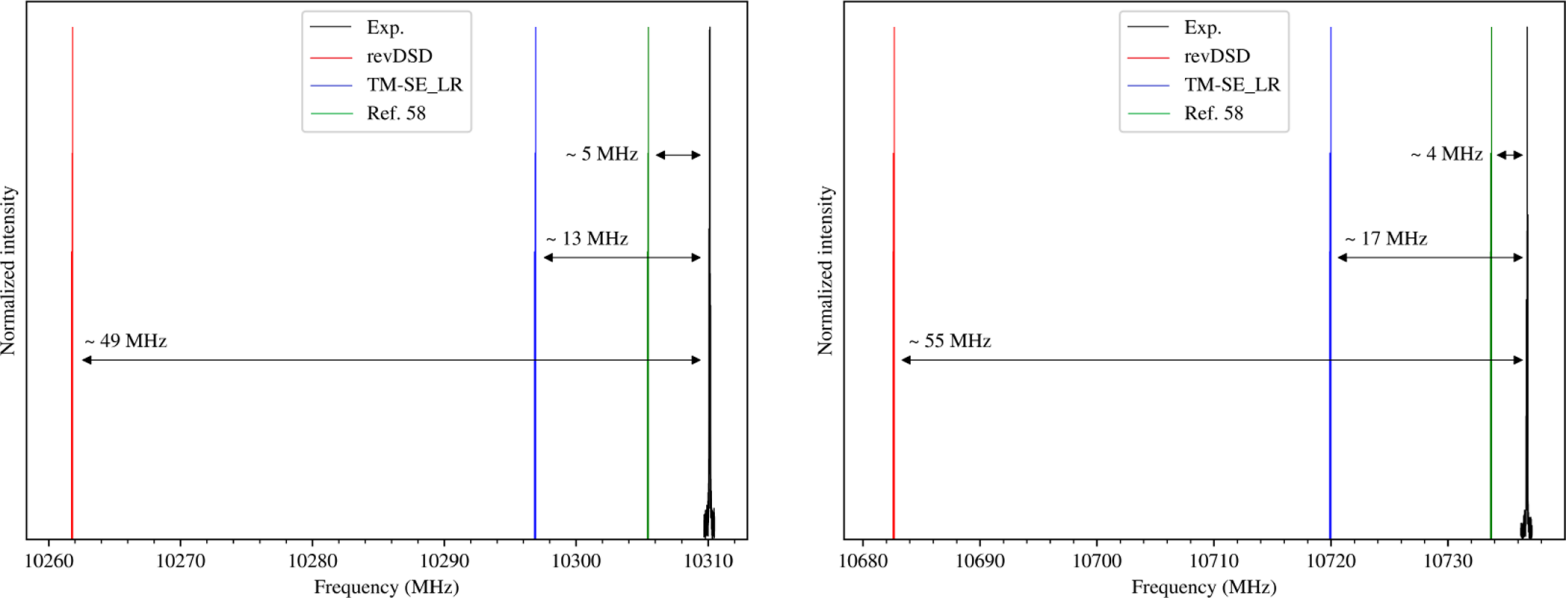
Comparison between the experimental spectrum (Exp.) and different simulations (revDSD, TM-SE_LR, and “cheap” scheme from ref (58)) for both isomers of PMI. The 4_1,4_ ← 3_1,3_ transition of *E*-PMI (left panel) and the 4_0,4_ ← 3_0,3_ transition of *Z*-PMI (right panel) have been chosen as examples ().

The comparison is shown in Figure 4 by means of two selected rotational transitions, one for *E*-PGIM and one for *Z*-PGIM. It is apparent that the TM-SE_LR approach leads to a significant improvement with respect to the starting revDSD level; indeed, the error associated to the transition frequency decreases by about three times. For the sake of completeness, the prediction based on the “cheap” composite scheme (from ref (58): “best theo” column of Table 1) has also been considered. It is noted that the latter (green stick spectra in Figure 4) is the most accurate; however, such a good agreement comes at the price of a much greater computational cost. In fact, the “cheap” composite scheme^22^ is based on the CCSD(T) method. Furthermore, for the specific case of PGIM, the latter approach showed an extraordinarily good agreement. Therefore, this example confirms that the TM-SE_LR approach is able to provide a remarkable accuracy in rotational spectral predictions, without increasing the computational effort with respect to revDSD. It is thus more than suitable for guiding and supporting experimental studies.

### Structure of 3-Phenyl-2-propynenitrile and Its Isomers

**Figure 5:**
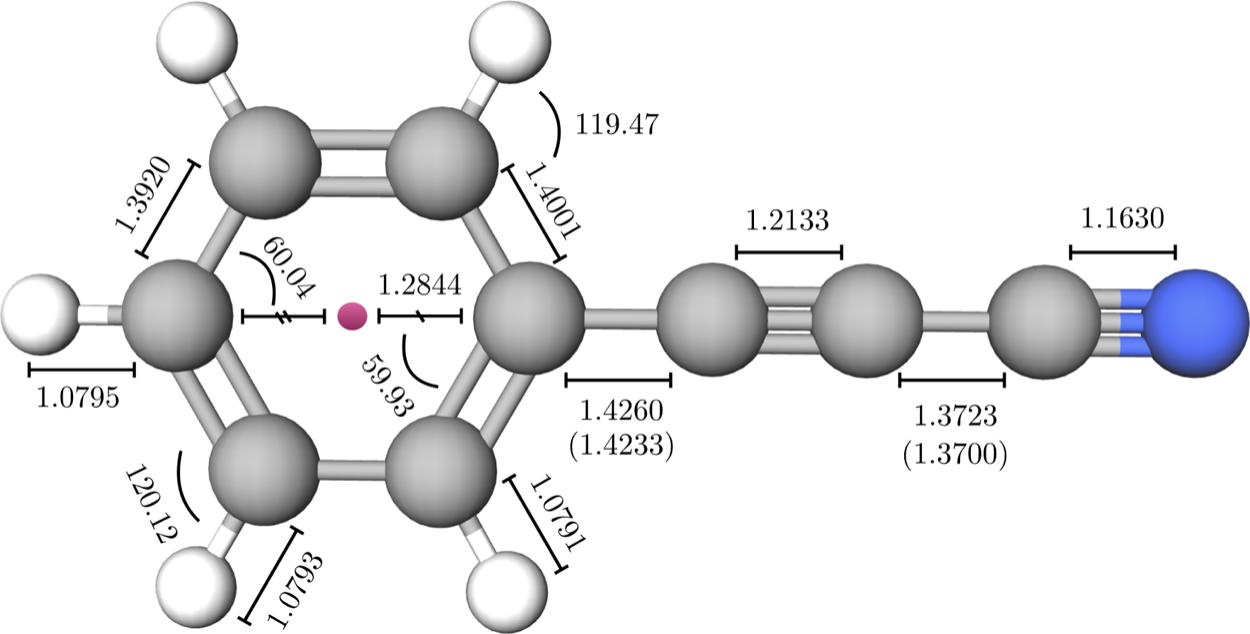
Structural parameters of 3-phenyl-2-propynenitrile obtained with the TM-SE and TM-SR_LR approaches. The two values in parentheses refer to LR-corrected C–C bond distances. All bond lengths are in Å, and angles are in degrees.

During the preparation of this manuscript, we became aware of the spectroscopic characterization of 3-phenyl-2-propynenitrile (PPN).^75^ Since—as noted above—the TM-SE approach works extremely well for rather rigid molecules, we decided to apply it to the structural determination of PPN and its isomers, thus providing an example of extension of the TM-SE approach to a larger system. For PPN, the resulting structure is shown in Figure 5, with the corresponding rotational constants being collected in Table 3. To build the PPN molecule, three fragments have been actually employed in the TM-SE approach: the phenyl moiety from benzene, the −C≡C– group from acetylene, and the −CN frame from HCN. Within the TM-SE_LR approach, the LR correction has been applied to the two C–C linkage bonds. All details can be found in Table S4 of the Supporting Information.

**Table 3:** Comparison between Computed Equilibrium Rotational Constants (in MHz) and the SE Counterparts for 3-Phenyl-2-propynenitrile^a^

	*B*_e_				
	revDSD	TM-SE	TM-SE_LR	SE^b^	*B*_0_^exp^ [Δ*B*_vib_^B3^]
*A*	5681.4 (−0.35%)	5703.0 (+0.01%)	5702.0 (+0.01%)	5701.4	5659.722(15) [41.6]
*B*	568.5 (−0.41%)	569.9 (−0.16%)	570.7 (−0.02%)	570.8	569.582206(39) [1.2]
*C*	516.8 (−0.41%)	518.1 (−0.15%)	518.8 (−0.01%)	518.9	517.404488(37) [1.5]

a The relative differences with respect to SE(*B*_e_^SE^) are reported in parentheses.

b Ground-state rotational constants from ref (75) corrected for vibrational corrections at the B3 level.

**Figure 6:**
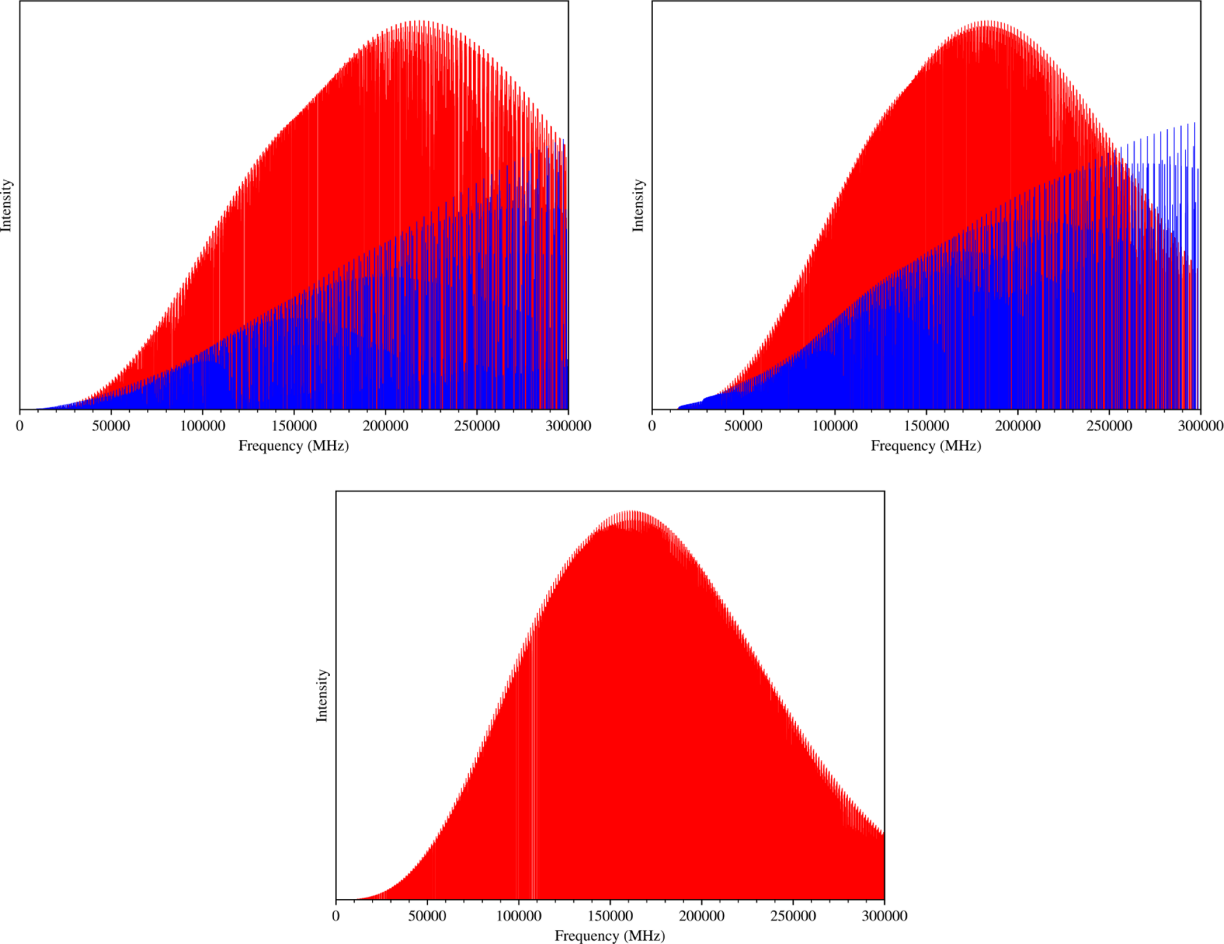
Simulation of the rotational spectrum of *o*- (top-left panel), *m*- (top-right panel), and *p*-CEB (bottom panel). The red and blue sticks represent the *a*- and *b*-type transitions, respectively.

From the inspection of Table 3, it is evident that—as expected—the TM-SE approach is able to predict accurate equilibrium rotational constants, which in turn means an accurate equilibrium structure. In particular, the results for the TM-SE_LR model are really impressive. Indeed, we note that going from revDSD to TM-SE leads to a reduction in the average relative error from 0.4 to 0.1%. Then, incorporation of the LR corrections further decreases the average relative deviation to 0.01%. In view of such a good performance, the TM-SE approach can be used to predict with great accuracy the rotational constants of different isomers of PPN: *o*-, *m*-, and *p*-cyanoethynylbenzene (CEB; see Table 4). Based on the analysis carried out in this manuscript and—in particular—for PPN, we expect that the equilibrium rotational constants issued from the TM-SE_LR approach have a relative accuracy well within 0.05%. Using the ground-state rotational constants of Table 4, the rotational spectra of *o*-, *m*-, and *p*-CEB have been simulated and are shown in Figure 6. For all the three molecules, the *a*-type transitions are the most intense and reach the maximum intensity in the millimeter-wave region, between 170 and 220 GHz. In this frequency region, the accuracy of the predicted rotational transitions is expected to range between 200 and 400 MHz, also accounting for the uncertainties affecting the computed centrifugal distortion constants (which are required for spectral predictions).

**Table 4:** Prediction of Rotational Constants (in MHz) of *o*-, *m*-, and *p*-cyanoethynylbenzene

	*B*_e_ (revDSD)	*B*_e_ (TM-SE)	*B*_e_ (TM-SE_LR)	Δ*B*_vib_	*B*_0_ (TM-SE_LR)^a^
o-CEB					
A	2025.1	2031.0	2033.00	4.66	2028.34
B	1330.2	1334.0	1335.48	6.11	1329.37
C	802.9	805.1	806.01	3.23	802.78
m-CEB					
A	2707.1	2715.4	2717.55	10.44	2707.11
B	906.6	909.0	910.16	3.47	906.69
C	679.1	681.0	681.81	2.80	679.01
p-CEB					
A	5673.0	5693.2	5693.19	45.85	5647.34
*B*	708.5	710.3	711.29	2.19	709.10
*C*	629.9	631.5	632.29	2.31	629.98

a *B*_e_(TM-SE_LR) constants augmented by B3 vibrational corrections.

## Conclusions

A benchmark study based on the availability of accurate semi-experimental equilibrium rotational constants for 21 molecular species allowed us to demonstrate the reliability, robustness, and accuracy of a “Lego brick” approach, the so-called TM-SE approach. For the selected molecules, each species has been seen as formed by two fragments, the “Lego bricks”, for which accurate semi-experimental structures are available. Accounting, at the rev-DSD-PBEP86-D3/jun-cc-pVTZ level, for the modifications taking place when moving from the isolated fragments to the molecular species of interest leads to the definition of the TM-SE equilibrium structure. A further improvement has been obtained by correcting the inter-fragment parameters with the linear regression corrective terms available for rev-DSD-PBEP86-D3/jun-cc-pVTZ. The resulting structure has been denoted as TM-SE_LR. For the TM-SE and TM-SE_LR equilibrium rotational constants, the average relative deviation with respect to accurate semi-experimental equilibrium rotational constants has been found to be ∼0.3 and ∼0.2%, respectively. According to the thorough investigation of the equilibrium geometries of 7 from the 21 molecules, these discrepancies reflect structural differences of about 0.001 Å for bond lengths and 0.1° for angles.

In conclusion, since the only quantum-chemical calculations required by the TM-SE and TM-SE_LR models are geometry optimizations at the rev-DSD-PBEP86-D3/jun-cc-pVTZ level, they are low-cost approaches able to provide an accuracy close to that of the most sophisticated composite approaches based on the coupled-cluster ansatz. Therefore, they are very promising tools for the accurate structural characterizations of medium- to large-sized molecular systems, such as the building blocks of biological molecules. Interestingly, test computations on a reduced dataset showed that the application of the TM-SE approach to B3-optimized geometries nearly doubles the relative deviations from the semi-experimental equilibrium rotational constants, thus implying a limited worsening in the structural parameters with respect to the models discussed above.

Finally, the TM-SE(_LR) approach is also suited well for unstable species of medium to large dimensions, which are of current interest in many different fields, ranging from astrochemistry to biochemistry, and whose structures can be hardly determined by experiments. Furthermore, our test on 3-phenyl-2-propynenitrile points out that the TM-SE(_LR) approach can be reliably extended to more than two “Lego bricks”, while the availability of LR corrections for bond lengths other than the C–C bond ones opens to its application to a wider range of molecular species.

## Supporting Information Available

Table S1. Template molecules: revDSD and semi-experimental equilibrium structures. Table S2: comparison between the semi-experimental and computed (revDSD, TM-SE, and TM-SE_LR) values of the structural parameters of vinylacetylene, vinyl cyanide, (*cis*, *trans*)-acrolein, *trans*-1,3-butadiene, *trans*-glyoxal, and *Z*-propargylimine. Table S3: computed equilibrium rotational constants and the SE counterparts together with the ground-state rotational constants and the corresponding vibrational corrections for the molecules of the dataset. Table S4: computed and template values of the structural parameters of 3-phenyl-2-propynenitrile and its isomers (*o*-, *m*-, and *p*-cyanoethynylbenzene) (PDF)

## Footnotes

a Note that for a given isotopic species, the number of rotational constants depends on the rotational symmetry. At most, there are three of them in the case of asymmetric rotors.
