# Supplementary material for: Extending the Applicability of the Semi-experimental Approach by Means of “Template Molecule” and “Linear Regression” Models on Top of DFT Computations
Source: J Phys Chem A. 2021 Nov 9;125(45):9904–16. doi: 10.1021/acs.jpca.1c07828 (PMC8607424; doi:10.1021/acs.jpca.1c07828)
Supplement: Supplementary file 1 — jp1c07828_si_001.pdf [file jp1c07828_si_001.pdf]

Supporting Information: Extending the Applicability of the Semi-Experimental Approach by Means of the 'Template Molecule' and 'Linear Regression' Models on Top of DFT Computations

Alessio Melli,[†,‡,¶] Francesca Tonolo,[†,‡,¶] Vincenzo Barone,[*,†] and Cristina Puzzarini[*,‡]

†*Scuola Normale Superiore, Piazza dei Cavalieri 7, 56126 Pisa, Italy*

‡*Dipartimento di Chimica "Giacomo Ciamician", Università di Bologna, Via Selmi 2, 40126 Bologna, Italy*

¶A.M. and F.T. contributed equally to this paper.

E-mail: vincenzo.barone@sns.it; cristina.puzzarini@unibo.it

Table S1: Template molecules: revDSD and semi-experimental equilibrium structures. Distances in Å and angles in degrees.

Fragment	Parameters		
	Name	revDSD[a]	Semiexp [Err.]
Methanimine	r(NH)	1.0211	1.0195[3]
	r(CN)	1.2721	1.2709[1]
	∠(CNH)	110.37	110.35[4]
	r(HcC)[b]	1.0932	1.092[3]
	∠(HcCN)[b]	124.47	123.7[5]
	r(HtC)[b]	1.0887	1.084[3]
	∠(HtCN)[b]	118.72	119.3[5]
Formaldehyde	r(CO)	1.2072	1.2047[1]
	r(CH)	1.1041	1.1003[1]
	∠(HCO)	121.78	121.65[1]
HCN	r(HC)	1.0674	1.0651[1]
	r(CN)	1.1575	1.1533[1]
Acetylene	r(CC)	1.2062	1.2036[1]
	r(CH)	1.0639	1.0611[1]
Ethene	r(CC)	1.3317	1.3311[1]
	r(CH)	1.0835	1.0807[1]
	∠(HCC)	121.50	121.42[1]
	∠(HCH)	117.01	117.16[1]
Acetonitrile	r(CN)	1.1598	1.1554[3]
	r(CC)	1.4614	1.4586[3]
	r(CH)	1.0897	1.0865[1]
	∠(HCC)	109.90	109.85[1]
Benzene	r(CC)	1.3939	1.3916[1]
	r(CH)	1.0837	1.0799[1]

[a] revDSD: revDSD-PBEP86-D3(BJ)/jun-cc-pVTZ.
[b] "c" e "t" refer to the *cis* and *trans* hydrogen, respectively.

Table S2: Comparison between the semi-experimental and the computed (revDSD, TM-SE and TM-SE_LR) values of the structural parameters of vinylacetylene, vinylcyanide, (*cis*,*trans*)-acrolein, *trans*-1,3-butadiene, *trans*-glyoxal and *Z*-propargylimine. Distances in Å and angles in degrees.

ZMAT	Parameters[a]			
	Name	revDSD[b]	Semiexp [Err.]	TM-SE (TM-SE_LR)
**VAC**				
H	R1	1.0637	1.0617[4]	1.0609
C 1 R1	R4	1.2111	1.2072[4]	1.2085
X 2 1.5 1 90.	R7	1.4285	1.4267[4]	1.4285 (1.4258)
X 3 1.5 2 90. 1 0.	R8	1.0852	1.0819[4]	1.0824
C 2 R4 3 90. 4 T2	R9	1.3387	1.3381[4]	1.3381
X 5 1.5 2 90. 3 180.	R10	1.0823	1.0798[4]	1.0795
X 6 1.5 5 90. 2 0.	R11	1.0828	1.0798[4]	1.0800
C 5 R7 6 90. 7 T5	A7	116.37	116.60[20]	116.37
H 8 R8 5 A7 6 90.	A8	119.95	120.07[20]	119.95
C 8 R9 9 A8 5 180.	A9	120.61	120.51[20]	120.61
H 10 R10 8 A9 9 0.	A10	121.33	121.21[20]	121.33
H 10 R11 8 A10 9 180.	T2	-179.43	-179.21[20]	-179.43
	T5	-178.50	-178.48[20]	-178.50
**VC**				
C	R1	1.3361	1.3351[4]	1.3355
C 1 R1	R2	1.4336	1.4315[4]	1.4336 (1.4309)
C 2 R2 1 A1	R3	1.1627	1.1584[4]	1.1586
X 3 1.5 2 90.0 1 180.0	R4	1.0823	1.0797[4]	1.0796
X 4 1.5 3 90.0 2 180.0	R5	1.0819	1.0785[4]	1.0791
N 3 R3 4 A2 5 0.0	R6	1.0831	1.0800[4]	1.0804
H 1 R4 2 A3 3 0.0	A1	122.29	122.03[7]	122.29
H 1 R5 2 A4 3 180.0	A2	88.97	89.14[7]	88.97
H 2 R6 1 A5 7 180.0	A3	121.44	121.31[7]	121.44
	A4	120.43	120.48[7]	120.43
	A5	121.54	121.66[7]	121.54

(continued)

(continued)

ZMAT	Parameters[a]			
	Name	revDSD[b]	Semiexp [Err.]	TM-SE (TM-SE_LR)
trans-**ACR**				
C	R1	1.0853	1.0827[2]	1.0825
H 1 R1	R2	1.0824	1.0792[2]	1.0796
H 1 R2 2 A1	R3	1.3369	1.3356[2]	1.3362
C 1 R3 3 A2 2 180.	R4	1.0841	1.0815[1]	1.0813
H 4 R4 1 A3 3 0.	R5	1.4732	1.4700[1]	1.4732 (1.4702)
C 4 R5 1 A4 3 180.	R6	1.1085	1.1049[2]	1.1047
H 6 R6 4 A5 1 0.	R7	1.2139	1.2105[1]	1.2114
O 6 R7 7 A6 4 180.	A1	117.29	117.48[2]	117.29
	A2	122.14	122.09[2]	122.14
	A3	122.57	122.76[2]	122.57
	A4	120.38	120.22[1]	120.38
	A5	114.86	115.02[2]	114.86
	A6	120.94	120.99[2]	120.94
cis-**ACR**				
C	R1	1.0838	1.0810[5]	1.0810
H 1 R1	R2	1.0824	1.0791[3]	1.0796
H 1 R2 2 A1	R3	1.3362	1.3359[4]	1.3356
C 1 R3 3 A2 2 180.	R4	1.0848	1.0814[3]	1.0821
H 4 R4 1 A3 3 0.	R5	1.4844	1.4818[4]	1.4844 (1.4814)
C 4 R5 1 A4 3 180.	R6	1.1060	1.1020[3]	1.1022
H 6 R6 4 A5 1 180.	R7	1.2142	1.2101[3]	1.2117
O 6 R7 4 A6 1 0.	A1	118.34	118.57[6]	118.34
	A2	121.62	121.59[5]	121.62
	A3	121.37	121.59[4]	121.37
	A4	121.62	121.28[3]	121.62
	A5	115.49	115.82[4]	115.49
	A6	124.31	123.88[3]	124.31

(continued)

(continued)

ZMAT	Parameters[a]			
	Name	revDSD[b]	Semiexp [Err.]	TM-SE (TM-SE_LR)
**BD**				
X	R1	0.7286	0.7274[1]	0.7286 (0.7271)
X 1 1.5	R2	1.3392	1.3380[2]	1.3386
C 1 R1 2 90.	R3	1.0870	1.0842[1]	1.0842
C 1 R1 2 90. 3 180.	R4	1.0824	1.0796[1]	1.0797
C 3 R2 1 A1 2 180.	R5	1.0846	1.0817[1]	1.0819
C 4 R2 1 A1 2 0.	A1	123.74	123.54[1]	123.74
H 3 R3 1 A2 2 0.	A2	116.65	116.65[2]	116.65
H 4 R3 1 A2 2 180.	A3	121.54	121.45[1]	121.54
H 5 R4 3 A3 1 180.	A4	121.08	120.96[1]	121.08
H 6 R4 4 A3 1 180.				
H 5 R5 3 A4 1 0.				
H 6 R5 4 A4 1 0.				
**GL**				
X	R1	0.7602	0.75727[19]	0.7602 (0.7585)
X 1 1.5	R2	1.1040	1.10071[26]	1.1002
C 1 R1 2 90.	R3	1.2086	1.20450[27]	1.2061
C 1 R1 2 90. 3 180.	A1	115.17	115.251[24]	115.17
H 3 R2 1 A1 2 0.	A2	121.36	121.277[31]	121.36
H 4 R2 1 A1 2 180.				
O 4 R3 1 A2 2 0.				
O 3 R3 1 A2 2 180.				

(continued)

(continued)

ZMAT	Parameters[a]			
	Name	revDSD[b]	Semiexp [Err.]	TM-SE (TM-SE_LR)
***Z*-PGIM**				
H	R1	1.0644	1.059[1]	1.0616
C 1 R1	R2	1.2106	1.207[3]	1.2080
X 2 1.5 1 90.	R3	1.4393	1.436[2]	1.4393 (1.4366)
X 3 1.5 2 90. 1 180.0	R4	1.0887	1.0840[c]	1.0840
C 2 R2 3 A1 4 0.0	R5	1.2786	1.272[3]	1.2774
X 5 1.5 2 90. 3 180.0	R6	1.0222	1.021[2]	1.0206
X 6 1.5 5 90. 2 0.0	A1	89.62	89.9[8]	89.62
C 5 R3 6 A2 7 180.0	A2	89.77	90.0[9]	89.77
H 8 R4 5 A3 6 0.0	A3	116.27	116.3[4]	116.27
N 8 R5 9 A4 6 180.0	A4	118.03	118.03[c]	118.03
H 10 R6 8 A5 5 0.0	A5	109.99	109.99[c]	109.99

[a] All the bond lenghts are in Angstrom. All the angles are in degrees.

[b] revDSD: revDSD-PBEP86-D3(BJ)/jun-cc-pVTZ.

[c] Fixed at the TM-SE value.

Table S3: Computed equilibrium rotational constants and the SE counterparts together with the ground-state rotational constants and the corresponding vibrational corrections for the molecules of the dataset. All values in MHz.

	B_e					B_0^{exp} [ΔB_{vib}^{B3}]
	revDSD	TM-SE	TM-SE_LR	TM-SE_LR+corr	SE	
Z-PGIM						
A	54707.3	54894.9	54904.5	54690.0	55040.8	54640.1468(45) [400.6]
	(−0.61%)	(−0.27%)	(−0.25%)	(−0.64%)		
B	4850.0	4858.9	4867.2	4873.9	4883.2	4862.362758(60) [20.9]
	(−0.68%)	(−0.50%)	(−0.33%)	(−0.19%)		
C	4455.1	4463.8	4470.8	4475.1	4485.2	4458.249970(55) [27.0]
	(−0.67%)	(−0.48%)	(−0.32%)	(−0.23%)		
E-PGIM						
A	63274.6	63397.5	63412.3	63134.1	63476.8	63099.2207(22) [377.6]
	(−0.32%)	(−0.12%)	(−0.10%)	(−0.54%)		
B	4752.0	4761.0	4768.9	4775.2	4787.1	4766.557614(55) [20.5]
	(−0.73%)	(−0.55%)	(−0.38%)	(−0.25%)		
C	4420.0	4428.4	4435.4	4439.4	4451.3	4425.560983(58) [25.7]
	(−0.70%)	(−0.51%)	(−0.36%)	(−0.27%)		
Z-CMI						
A	54326.8	54527.8	54537.2	54330.0	54644.0	54193.405(32) [450.6]
	(−0.58%)	(−0.21%)	(−0.20%)	(−0.57%)		
B	5057.5	5070.7	5079.3	5086.7	5095.8	5073.86584(15) [22.0]
	(−0.75%)	(−0.49%)	(−0.32%)	(−0.18%)		
C	4626.7	4639.3	4646.6	4651.2	4661.0	4632.38905(14) [28.7]
	(−0.74%)	(−0.47%)	(−0.31%)	(−0.21%)		
E-CMI						
A	62939.3	63084.0	63100.1	62830.2	63101.5	62700.392(22) [401.1]
	(−0.26%)	(−0.03%)	(−0.002%)	(−0.43%)		
B	4951.0	4966.8	4975.1	4982.0	4994.8	4972.04534(22) [22.7]
	(−0.88%)	(−0.56%)	(−0.39%)	(−0.26%)		
C	4590.0	4604.3	4611.5	4615.9	4628.3	4600.29561(23) [28.0]
	(−0.83%)	(−0.52%)	(−0.36%)	(−0.27%)		

(continued)

(continued)

	B_e					B_0^{exp} [ΔB_{vib}^{B3}]
	revDSD	TM-SE	TM-SE_LR	TM-SE_LR+corr	SE	
Z-PMI						
A	5229.2	5247.6	5247.8	5246.0	5242.9	5200.81278(16) [42.0]
	(−0.26%)	(+0.09%)	(+0.09%)	(+0.06%)		
B	1550.6	1554.0	1556.0	1557.2	1558.8	1548.969349(92) [9.9]
	(−0.53%)	(−0.31%)	(−0.18%)	(−0.11%)		
C	1196.0	1198.9	1200.1	1200.8	1201.9	1194.842313(78) [7.1]
	(−0.50%)	(−0.25%)	(−0.15%)	(−0.10%)		
E-PMI						
A	5241.9	5260.2	5260.3	5258.5	5257.5	5217.29202(11) [40.2]
	(−0.30%)	(+0.05%)	(+0.05%)	(+0.02%)		
B	1567.1	1570.4	1572.3	1573.6	1574.8	1565.283633(28) [9.5]
	(−0.49%)	(−0.28%)	(−0.16%)	(−0.08%)		
C	1206.4	1209.4	1210.5	1211.2	1212.0	1204.540307(14) [7.4]
	(−0.46%)	(−0.21%)	(−0.12%)	(−0.07%)		
CF						
A	66979.5	67294.3	67313.0	67018.0	67566.7	67469.6749(29) [97.0]
	(−0.87%)	(−0.40%)	(−0.38%)	(−0.81%)		
B	4989.9	5004.8	5014.4	5021.3	5024.5	5010.18856(34) [14.3]
	(−0.69%)	(−0.39%)	(−0.20%)	(−0.06%)		
C	4643.9	4658.3	4666.7	4671.3	4676.6	4656.60175(29) [20.0]
	(−0.70%)	(−0.39%)	(−0.21%)	(−0.11%)		
PGCN						
A	19652.8	19736.2	19751.8	19662.4	19848.6	19820.080(70) [28.6]
	(−0.99%)	(−0.57%)	(−0.49%)	(−0.94%)		
B	2902.8	2912.4	2916.1	2922.8	2913.4	2909.6062(12) [3.8]
	(−0.36%)	(−0.03%)	(+0.09%)	(+0.32%)		
C	2569.3	2578.1	2581.2	2584.9	2580.5	2573.2123(12) [7.3]
	(−0.43%)	(−0.10%)	(+0.03%)	(+0.17%)		

(continued)

(continued)

	B_e					B_0^{exp} [ΔB_{vib}^{B3}]
	revDSD	TM-SE	TM-SE_LR	TM-SE_LR+corr	SE	
VC						
A	50115.1	50250.1	50262.7	50071.7	49893.3	49850.6974(20) [42.6]
	(+0.44%)	(+0.72%)	(+0.74%)	(+0.36%)		
B	4955.5	4967.0	4975.5	4982.8	4986.5	4971.163651(24) [15.3]
	(−0.62%)	(−0.39%)	(−0.22%)	(−0.07%)		
C	4509.6	4520.2	4527.4	4531.9	4533.3	4513.877260(25) [19.4]
	(−0.52%)	(−0.29%)	(−0.13%)	(−0.03%)		
MN						
A	20674.4	20746.9	20784.8	20690.6	20916.4	20882.77323(53) [33.6]
	(−1.16%)	(−0.81%)	(−0.63%)	(−1.08%)		
B	2934.7	2942.2	2949.7	2956.4	2945.8	2942.304967(75) [3.5]
	(−0.38%)	(−0.12%)	(+0.13%)	(+0.36%)		
C	2611.6	2618.5	2625.1	2628.9	2623.8	2616.727165(66) [7.1]
	(−0.47%)	(−0.20%)	(+0.05%)	(+0.19%)		
E-AIM						
A	46182.1	46286.3	46341.1	46254.9	46252.4	45773.628(18) [478.8]
	(−0.15%)	(+0.07%)	(+0.19%)	(+0.01%)		
B	4568.6	4573.0	4580.0	4584.3	4587.8	4560.916(4) [26.8]
	(−0.42%)	(−0.32%)	(−0.17%)	(−0.07%)		
C	4157.3	4161.8	4168.1	4170.9	4174.4	4148.242(3) [26.1]
	(−0.41%)	(−0.30%)	(−0.15%)	(−0.08%)		
Z-AIM						
A	44166.3	44307.8	44361.7	44286.8	44215.4	43759.52(18) [455.9]
	(−0.11%)	(+0.21%)	(+0.33%)	(+0.16%)		
B	4573.3	4577.6	4584.5	4588.8	4593.9	4564.581(39) [29.4]
	(−0.45%)	(−0.36%)	(−0.21%)	(−0.11%)		
C	4144.1	4148.9	4155.1	4157.9	4162.1	4134.423(30) [27.6]
	(−0.43%)	(−0.32%)	(−0.17%)	(−0.10%)		

(continued)

(continued)

	B_e					B_0^{exp} [ΔB_{vib}^{B3}]
	revDSD	TM-SE	TM-SE_LR	TM-SE_LR+corr	SE	
STY						
A	5195.5	5213.8	5214.0	5211.9	5205.8	5163.385(11) [42.4]
	(−0.20%)	(+0.15%)	(+0.16%)	(+0.12%)		
B	1546.9	1550.1	1552.1	1553.4	1555.1	1545.1699(7) [9.9]
	(−0.53%)	(−0.32%)	(−0.20%)	(−0.11%)		
C	1192.0	1194.9	1196.0	1196.7	1198.3	1191.2240(7) [7.1]
	(−0.52%)	(−0.28%)	(−0.19%)	(−0.13%)		
VAC						
A	50611.7	50741.1	50751.6	50552.9	50602.3	50300.158(55) [302.2]
	(+0.02%)	(+0.27%)	(+0.30%)	(−0.10%)		
B	4730.2	4738.2	4745.9	4752.6	4759.4	4744.94254(22) [14.4]
	(−0.61%)	(−0.45%)	(−0.28%)	(−0.14%)		
C	4325.9	4333.5	4340.1	4344.2	4350.1	4329.77304(23) [20.3]
	(−0.56%)	(−0.38%)	(−0.23%)	(−0.14%)		
BAL						
A	5256.4	5275.8	5276.0	5274.1	5273.0	5234.364365(54) [38.6]
	(−0.31%)	(+0.05%)	(+0.06%)	(+0.02%)		
B	1565.3	1569.1	1571.1	1572.4	1573.7	1564.274377(22) [9.4]
	(−0.53%)	(−0.29%)	(−0.16%)	(−0.08%)		
C	1206.1	1209.4	1210.6	1211.3	1212.1	1204.681915(16) [7.4]
	(−0.49%)	(−0.22%)	(−0.12%)	(−0.07%)		
PA						
A	67635.2	67933.1	67949.0	67641.0	68074.6	68035.2557(13) [39.4]
	(−0.65%)	(−0.21%)	(−0.18%)	(−0.64%)		
B	4808.4	4819.4	4828.3	4834.6	4840.1	4826.22365(10) [13.8]
	(−0.65%)	(−0.43%)	(−0.24%)	(−0.11%)		
C	4489.2	4500.1	4507.9	4512.1	4518.8	4499.592240(99) [19.2]
	(−0.65%)	(−0.41%)	(−0.24%)	(−0.15%)		

(continued)

(continued)

	B_e					B_0^{exp} [ΔB_{vib}^{B3}]
	revDSD	TM-SE	TM-SE_LR	TM-SE_LR+corr	SE	
trans-ACR						
A	47786.8	47934.4	47996.9	47899.1	47857.9	47353.729(9) [504.1]
	(−0.15%)	(+0.16%)	(+0.29%)	(+0.09%)		
B	4660.4	4667.9	4675.5	4680.3	4685.0	4659.4894(4) [25.6]
	(−0.53%)	(−0.37%)	(−0.20%)	(−0.10%)		
C	4246.3	4253.7	4260.5	4263.7	4267.9	4242.7034(4) [25.2]
	(−0.51%)	(−0.33%)	(−0.18%)	(−0.10%)		
cis-ACR						
A	22938.3	23009.8	23010.0	22971.9	22896.4	22831.650(16) [64.7]
	(+0.18%)	(+0.50%)	(+0.50%)	(+0.33%)		
B	6228.5	6235.5	6250.2	6263.0	6295.8	6241.0470(29) [54.7]
	(−1.08%)	(−0.97%)	(−0.73%)	(−0.53%)		
C	4898.4	4906.0	4915.1	4921.3	4939.4	4902.2063(28) [37.2]
	(−0.84%)	(−0.68%)	(−0.50%)	(−0.37%)		
BN						
A	5678.3	5698.9	5698.9		5697.8	5655.26522(59) [42.5]
	(−0.34%)	(+0.02%)	(+0.02%)			
B	1546.7	1551.4	1553.1		1552.8	1546.875836(63) [5.9]
	(−0.39%)	(−0.09%)	(+0.02%)			
C	1215.6	1219.4	1220.5		1220.3	1214.404061(48) [5.9]
	(−0.38%)	(−0.07%)	(+0.02%)			
BD						
A	42069.0	42190.6	42239.2	42123.9	42127.1	41682.6577(21) [444.5]
	(−0.14%)	(+0.15%)	(+0.27%)	(−0.01%)		
B	4441.7	4445.0	4451.8	4459.7	4460.6	4433.5047(30) [27.1]
	(−0.43%)	(−0.35%)	(−0.20%)	(−0.02%)		
C	4017.5	4021.3	4027.3	4032.7	4034.3	4008.0423(60) [26.2]
	(−0.42%)	(−0.32%)	(−0.17%)	(−0.04%)		

(continued)

(continued)

	B_e					B_0^{exp} $[\Delta B_{vib}^{B3}]$
	revDSD	TM-SE	TM-SE_LR	TM-SE_LR+corr	SE	
GL						
A	55888.5	56092.7	56183.9	55973.2	55955.7	55290.6121(0510) [665.1]
	(−0.12%)	(+0.25%)	(+0.41%)	(+0.03%)		
B	4781.5	4792.6	4802.1	4811.6	4821.0	4798.0371(69) [23.0]
	(−0.82%)	(−0.59%)	(−0.40%)	(−0.20%)		
C	4404.7	4415.4	4423.9	4430.7	4439.0	4416.8983(69) [22.2]
	(−0.78%)	(−0.54%)	(−0.34%)	(−0.19%)		

Table S4: Computed and template values of the structural parameters of 3-phenyl-2-propynenitrile and its isomers (*o*-, *m*-, and *p*-cyanoethynylbenzene). Distances in Å and angles in degrees.

ZMAT	Parameters[a]		
	Name	revDSD[b]	TM-SE (TM-SE_LR)
**PPN**			
X	R1	1.2889	1.2844
X 1 1.5	R2	1.4260	1.4260 (1.4233)
C 1 1.5 2 90.	R3	1.2159	1.2133
C 1 R1 2 90. 3 180.	R4	1.0833	1.0795
X 4 1.5 1 90. 2 0.	R5	1.4024	1.4001
X 3 1.5 1 90. 2 0.	R6	1.3943	1.3920
C 3 R2 6 90. 2 180.	R7	1.0829	1.0791
X 7 1.5 3 90. 6 90.	R8	1.0831	1.0793
C 7 R3 8 90. 3 180.	R9	1.3723	1.3723 (1.3700)
X 9 1.5 7 90. 8 0.	R10	1.1671	1.1630
H 4 R4 5 90. 2 180.	A1	59.93	59.93
C 3 R5 1 A1 2 -90.	A2	60.04	60.04
C 3 R5 1 A1 2 90.	A3	119.47	119.47
C 4 R6 1 A2 2 90.	A4	120.12	120.12
C 4 R6 1 A2 2 -90.			
H 12 R7 3 A3 1 180.			
H 13 R7 3 A3 1 180.			
H 14 R8 4 A4 1 180.			
H 15 R8 4 A4 1 180.			
C 9 R9 10 90. 8 180.			
X 20 1.5 9 90. 10 0.			
N 20 R10 21 90. 10 180.			

(continued)

(continued)

ZMAT	Parameters[a]		
	Name	revDSD[b]	TM-SE (TM-SE_LR)
***o*-CEB**			
H	R1	1.0643	1.0614
C 1 R1	R2	1.2100	1.2074
X 2 1.5 1 90.	R3	1.4280	1.4280 (1.4253)
C 2 R2 3 A1 1 180.	R4	1.4093	1.4070
X 4 1.5 2 90. 3 0.	R5	1.3981	1.3958
C 4 R3 5 A2 3 180.	R6	1.3896	1.3873
C 6 R4 4 A3 5 180.	R7	1.3947	1.3924
C 7 R5 6 A4 4 180.	R8	1.3900	1.3877
C 8 R6 7 A5 6 0.	R9	1.0825	1.0787
C 9 R7 8 A6 7 0.	R10	1.0831	1.0793
C 10 R8 9 A7 8 0.	R11	1.0828	1.0790
H 11 R9 10 A8 9 180.	R12	1.0826	1.0788
H 10 R10 9 A9 8 180.	R13	1.4343	1.4343 (1.4316)
H 9 R11 8 A10 7 180.	R14	1.1630	1.1588
H 8 R12 7 A11 6 180.	A1	90.62	90.62
C 7 R13 6 A12 4 0.	A2	90.22	90.22
X 16 1.5 7 90. 6 180.	A3	120.68	120.68
N 16 R14 17 A13 7 180.	A4	120.42	120.42
	A5	120.03	120.03
	A6	119.91	119.91
	A7	120.35	120.35
	A8	120.63	120.63
	A9	120.04	120.04
	A10	119.81	119.81
	A11	119.17	119.17
	A12	120.18	120.18
	A13	88.73	88.73

(continued)

(continued)

ZMAT	Parameters[a]		
	Name	revDSD[b]	TM-SE (TM-SE_LR)
***m*-CEB**			
H	R1	1.0642	1.0614
C 1 R1	R2	1.2102	1.2076
X 2 1.5 1 90.	R3	1.4313	1.4313 (1.4286)
C 2 R2 3 A1 1 180.	R4	1.3986	1.3963
X 4 1.5 2 90. 1 180.	R5	1.0822	1.0785
C 4 R3 5 A2 3 180.	R6	1.3964	1.3941
C 6 R4 4 A3 5 180.	R7	1.3994	1.3971
H 7 R5 6 A4 4 0.	R8	1.3907	1.3884
C 7 R6 6 A5 4 180.	R9	1.3910	1.3887
C 9 R7 7 A6 8 180.	R10	1.0828	1.0790
C 10 R8 9 A7 7 0.	R11	1.0828	1.0790
C 11 R9 10 A8 9 0.	R12	1.0825	1.0787
H 12 R10 6 A9 4 0.	R13	1.4360	1.4360 (1.4333)
H 11 R11 12 A10 6 180.	R14	1.1628	1.1586
H 10 R12 11 A11 12 180.	A1	90.11	90.11
C 9 R13 7 A12 8 0.	A2	89.91	89.91
X 16 1.5 9 90. 7 0.	A3	120.12	120.12
N 16 R14 17 A13 9 180.	A4	120.05	120.05
	A5	119.78	119.78
	A6	120.63	120.63
	A7	119.37	119.37
	A8	120.42	120.42
	A9	119.17	119.17
	A10	119.81	119.81
	A11	120.91	120.91
	A12	119.58	119.58
	A13	90.19	90.19

(continued)

(continued)

ZMAT	Parameters[a]		
	Name	revDSD[b]	TM-SE (TM-SE_LR)
***p*-CEB**			
X	R1	1.2926	1.2881
X 1 1.5	R2	1.4304	1.4304 (1.4277)
C 1 1.5 2 90.	R3	1.2104	1.2078
C 1 R1 2 90. 3 180.	R4	1.4347	1.4347 (1.4320)
X 4 1.5 1 90. 2 0.	R5	1.4023	1.4001
X 3 1.5 1 90. 2 0.	R6	1.3997	1.3974
C 3 R2 6 90. 2 180.	R7	1.0825	1.0787
X 7 1.5 3 90. 6 90.	R8	1.0826	1.0788
C 7 R3 8 90. 3 180.	R9	1.0642	1.0614
X 9 1.5 7 90. 8 0.	R10	1.1631	1.1589
C 4 R4 5 90. 2 180.	A1	59.72	59.72
C 3 R5 1 A1 2 TM90	A2	60.08	60.08
C 3 R5 1 A1 2 90.	A3	119.38	119.38
C 4 R6 1 A2 2 90.	A4	119.70	119.70
C 4 R6 1 A2 2 -90.			
H 12 R7 3 A3 1 180.			
H 13 R7 3 A3 1 180.			
H 14 R8 4 A4 1 180.			
H 15 R8 4 A4 1 180.			
H 9 R9 10 90. 8 180.			
X 11 1.5 4 90. 5 90.			
N 11 R10 21 90. 4 180.			

[a] All the bond lenghts are in Angstrom. All the angles are in degrees.

[b] revDSD: revDSD-PBEP86-D3(BJ)/jun-cc-pVTZ.
